# Circuitous Ways of EWS::FLI1 Using Circular RNA ZNF609 to Evade Translational Repression by miR-145 in Ewing’s Sarcoma

**DOI:** 10.3390/biomedicines14010129

**Published:** 2026-01-08

**Authors:** Aakash Koppula, Ahmed Abdelgawad, Brigette Romero, Victoria Beringer, Vijay Parashar, Mona Batish

**Affiliations:** 1 Department of Biological Sciences, University of Delaware, 15 Innovation Way, Newark, DE 19711, USA; aakash@udel.edu (A.K.); ahmedg@udel.edu (A.A.); parashar@udel.edu (V.P.); 2 Department of Medical and Molecular Sciences, University of Delaware, 15 Innovation Way, Newark, DE 19711, USA; bmromero@udel.edu (B.R.); vbering@udel.edu (V.B.)

**Keywords:** Ewing’s Sarcoma, EwS, EWS:: FLI1, circular RNA, circRNA, circZNF609, microRNA, miRNA, miR145

## Abstract

**Background:** Ewing’s sarcoma (EwS) is a pediatric bone and soft tissue cancer driven by the oncogenic fusion protein EWS::FLI1. Currently, EwS lacks targeted therapies, necessitating the identification of novel regulatory mechanisms. While the role of microRNAs and long non-coding RNAs has been explored in EwS, the presence and functional significance of circular RNAs (circRNAs) in EwS is not reported. This is the first study to report the presence and role of oncogenic circRNA, circZNF609 in EwS tumor progression. **Methods:** Expression of circZNF609 was validated in 5 different EwS cell lines using qPCR. Cellular localization of circZNF609 was identified using circFISH. Functional assays for proliferation, migration and apoptosis were performed in wild type and circZNF609 knocked down (KD) cell lines to confirm its oncogenic role. The impact of circZNF609 on EWS::FLI1 protein levels was confirmed using western blots, immunofluorescence, and polysome fractionation. Mechanistic insights were gained utilizing bioinformatic, dual-luciferase reporter assays, rescue experiments, and microscopy to identify and validate the circRNA-miRNA-mRNA regulatory axis. **Results:** We report the first identification of circZNF609 in EwS, demonstrating that its expression is EWS::FLI1-dependent. Functional analysis reveals that circZNF609 promotes cell proliferation and metastasis while inhibiting apoptosis. Mechanistically, circZNF609 acts as a molecular sponge for miR-145-5p. By sequestering this miRNA, circZNF609 prevents the translational repression of EWS::FLI1, thereby sustaining oncogenic signaling. **Conclusions:** These findings identify circZNF609 as a novel post-transcriptional regulator of EWS::FLI1 and establish its critical role in EwS pathogenesis. Our results suggest that targeting the circZNF609/miR-145-5p/EWS::FLI1 axis may offer a promising therapeutic strategy for EwS.

## 1. Introduction

Ewing’s sarcoma (EwS) is a bone and soft tissue cancer in children, with an incidence of approximately 2–4 cases per million, and it is associated with a very high mortality rate upon metastasis [1]. The molecular cause of EwS is a chromosomal rearrangement between the *EWSR1* gene on chromosome 22 and members of the *ETS* gene family (most commonly *FLI1*, in 90% of cases) on chromosome 11, resulting in the formation of the aberrant oncogenic fusion protein EWS::FLI1. EWS::FLI1 is known to function as an enhancer, transcription factor, and splicing mediator to initiate tumorigenic changes; however, the precise mechanisms by which this single, highly disordered oncogenic protein drives tumor progression remain incompletely understood [2,3]. The lack of structural information and the unstable nature of the fusion protein have made it challenging to develop drugs targeting EWS::FLI1, which would otherwise be an ideal therapeutic target. Although the EWSR1-FLI1 fusion has been recognized as the molecular cause of EwS for over 40 years, no targeted therapies are currently available. As a result, alternative approaches have focused on understanding the interacting partners and downstream effectors of EWS::FLI1, with most work to date emphasizing chromatin regulation [4], epigenetics [5], and protein partners [6]. However, the contribution of non-coding RNAs (ncRNAs) to Ewing’s sarcoma pathogenesis remains poorly understood. Given that EwS cells have a relatively stable genome [1], it is important to explore non-genetic regulatory factors that may contribute to EWS::FLI1-mediated transformation in order to identify novel therapeutic targets.

The majority of the human genome is transcribed into ncRNAs, whose regulatory roles are only beginning to be appreciated [7]. Among these, a newly recognized class of ncRNAs, circular RNAs (circRNAs), has been shown to play regulatory roles in various cancers, and their mechanisms of action are an emerging area of research [8,9]. CircRNAs are covalently closed RNA molecules with no free ends, typically formed by base pairing between repeat regions of long introns flanking exons, leading to back-splicing and the release of a circular molecule. While circRNAs can be derived from exons, introns, or a combination of both, exonic circRNAs are considered the most clinically relevant [10,11]. Although circRNAs were discovered about 40 years ago, they were long considered splicing errors until the last decade, when their roles in various physiological processes began to be elucidated [8]. The most studied function of circRNAs is their ability to act as microRNA (miRNA) sponges [12]. A growing body of literature has identified functional roles for circRNAs in the progression of many cancers [8,9]. Reducing their expression or blocking their interactions with targets has led to dramatic changes in cancer progression, suggesting that circRNAs may be promising therapeutic targets [9,13]. Furthermore, due to their resistance to exonucleases and their enrichment in extracellular vesicles (EVs), circRNAs are promising biomarkers for non-invasive and early diagnosis of many solid tumors [9]. Despite the recognition of multiple critical roles for circRNAs in various cancers, including osteosarcoma [13], there have been limited studies characterizing circRNAs in EwS cells or in EVs derived from EwS cells. While the roles of miRNAs and long ncRNAs in EwS oncogenesis have been previously studied [14,15,16], the expression and function of circRNAs in EwS remain unexplored. In this study, we focus on circZNF609, a known oncogenic circRNA previously reported in several cancers, including breast cancer and hepatocellular carcinoma [17,18]. CircZNF609 has also been reported to act as a sponge for miR-145, which has been extensively studied in EwS, as well as other miRNAs in several cancers [18,19]. Here, we report that circZNF609 is expressed in an EWS::FLI1-dependent manner in EwS and acts as a miR-145-5p sponge, thereby preventing miR-145-5p from binding to the 3′ UTR of EWS::FLI1. This relieves miR-145-5p-mediated suppression of EWS::FLI1 and contributes to EwS oncogenesis.

## 2. Materials and Methods

Cell lines and cloning: HEK 293T (ATCC, Manassas, VA, USA, CRL-3216) cell line was cultured in DMEM (Millipore Sigma, St. Louis, MO, USA, D6429) supplemented with 10% FBS (Millipore Sigma, St. Louis, MO, USA, F2442), penicillin/streptomycin (Millipore Sigma, St. Louis, MO, USA, P4333), and L-glutamine (Millipore Sigma, St. Louis, MO, USA, G7513). A673 inducible cell lines were a gift from Dr. Olivier Delattre, Institut Curie, and all other EwS cell lines were a gift from Dr. James Wells, Harvard Medical School. A673 inducible cells lines and 6647 cells were cultured in the above mentioned DMEM, further supplemented with HEPES (Millipore Sigma, St. Louis, MO, USA, H0887) and non-essential amino acids (Millipore Sigma, St. Louis, MO, USA, M7145). TC32 and A4573 cell lines were cultured in RPMI media (Millipore Sigma, St. Louis, MO, USA, R8758) supplemented with 10% FBS, penicillin/streptomycin, and HEPES. SKES-1 cells were cultured in McCoy’s 5A media (Millipore Sigma, St. Louis, MO, USA, M9309) supplemented with 10% FBS and penicillin/streptomycin. The human immortalized myoblast AB678 cell line was a gift from Dr. Myriam Gorospe, laboratory of Genetics and Genomics at National Institute of Aging at NIH. AB678 were cultured in 1:1 HAMS F-10 (Cytiva, Marlborough, MA, USA, SH30025.01) and Promocell skeletal muscle cell growth medium (PromoCell Inc., Heidelberg, Germany, C-23060) supplemented with 20% FBS and 1% penicillin/streptomycin. For cloning, shRNA targeting the BSJ of circZNF609 was cloned into the pLKO.1-TRC cloning vector (Addgene Plasmid 10878) and transfected into 293T EBNA cells for virus production. The shRNA sequences used are provided in Table S1. The scrambled shRNA sequence was cloned into pLKO.1-TRC cloning vector and was used as a negative control. A673 inducible and TC32 cells were transduced with the virus, and stable cell lines were selected via puromycin (Gibco, Waltham, MA, USA, A11138-03). All cells were cultured at 37 °C with 5% CO_2_. For imaging studies, all cells were grown on 0.1% gelatin (Bio-Rad, Hercules, CA, USA, 170-6537)-coated coverslips in 100 cm^2^ Petri dishes until 70–80% confluent before fixing in 4% PFA (Millipore Sigma, St. Louis, MO, USA, F8775) and permeabilizing in 70% ethanol for hybridization.

Probe synthesis and purification: Sets of 45–50 linear oligonucleotide probes, each 20 nucleotides in length, were designed to be complementary to specific regions of ZNF609 and circZNF609 RNA molecules, along with an amino group on their 3′ ends (Biosearch Technologies, Novato, CA, USA) to generate PC and PL probes for the ZNF609. The probes were pooled in equimolar concentrations and conjugated with Texas Red [TR] (Invitrogen, Waltham, MA, USA, T6134) or Cy5 (GE Healthcare, Chicago, IL, USA, PA25001) fluorophores and purified by high-pressure liquid chromatography as previously described in [20]. The list of probe sequences used is provided in Supplementary Table S2.

In situ circFISH Hybridization: Coverslips with fixed adherent cells were washed with 2× saline sodium citrate solution (Ambion, Austin, TX, USA, AM9763) containing 20% formamide (Ambion, Austin, TX, USA, AM9342/44) and 2 mM ribonucleoside–vanadyl complex (New England Biolabs, Ipswich, MA, USA, S1402S), then cells were hybridized with probe sets in hybridization buffer containing 25 ng/μL of each probe set. Hybridization was performed overnight at 37 °C in a moist chamber. The next day, the coverslips were washed three times and stained with DAPI (Sigma, St. Louis, MO, USA, D9542) and mounted on glass slides for imaging. For circRNA-miRNA imaging, circFISH and miRNA detection probes (Qiagen, Hilden, Germany, 339350) were used to visualize the interaction between circZNF609 and miR-145-5p, following the protocol adapted by [21].

Fluorescence imaging and analysis: All images were captured with a 100× oil objective using a Nikon TiE Inverted epi Fluorescence microscope equipped with a pixis 1024b camera (Princeton Instruments, Trenton, NJ, USA). The images were obtained using Metamorph imaging software, version 7.8.13.0 (Molecular Devices, San Jose, CA, USA). Z-stack images were captured for each fluorescent wavelength at 2 sec exposures, for a total of 16 stacks, 0.2 mm apart. The compiled z-stack images were analyzed using an in-house-designed algorithm with MATLAB software, version 23.2.0.2365128 (R2023b) (MathWorks, Natick, MA, USA) that identifies spot-like signals in each image and determines their three-dimensional coordinates, then identifies spots that have a counterpart within a 250 nm distance in the other channel. Spots meeting this criterion are classified as co-localized. Each imaging experiment was performed to obtain at least 100 cell counts. The numbers present average molecules with errors indicating 95% confidence interval. The *p*-values were obtained using Student’s t-test.

qRT-PCR: Total RNA was extracted from cells lysed in Trizol (Sigma, St. Louis, MO, USA, T9424), and equal concentrations of RNA from different cells were used for cDNA synthesis using iScript Reverse Transcription Supermix (Bio-RAD, Hercules, CA, USA, 1708841), and gene expression was analyzed using iTaq Universal SYBR Green Supermix (Bio-RAD, Hercules, CA, USA, 1725124) according to the manufacturer’s protocol. List of primers is provided in Supplementary Table S3.

Immunofluorescence: For immunofluorescence, the permeabilized A673 and TC32 cells were incubated with blocking buffer followed by overnight incubation at 4 °C with primary antibodies. The subsequent day, another blocking step was performed, followed by incubation with a secondary antibody for 1 h at room temperature, and then followed by four to five washes with PBS. For imaging, the cells were mounted with DAPI containing mounting medium and imaged using a 100× oil objective in a Nikon TiE inverted fluorescence microscope equipped with a CCD Princeton Pixis 1024b camera. The images were acquired using Metamorph software. The corrected total cell fluorescence (CTCF) was calculated using ImageJ 1.54d (National Institutes of Health, Bethesda, MD, USA) [22], and the following formula was applied: CTCF = integrated density − (area of selected cell × mean fluorescence of background readings), as per [23]. A list of antibodies is provided in Supplementary Table S4.

Proliferation assay: Cell proliferation was performed using the 3-(4, 5-dimethylthiazol-2-yl)-2, 5-diphenyltetrazolium bromide (MTT) Cell Proliferation kit (Roche, Basel, Switzerland, 11465007001). After harvesting the cells, they were reseeded in 96-well plates with approximately 5000 cells/well with media and maintained at 37 °C in the CO_2_ incubator for 96 h. Cells were treated based on the manufacturer’s protocol at 0, 24, 48, 72, and 96 h timepoints using a plate reader.

Annexin V assay: Annexin V/Propidium Iodide staining was performed using Annexin V Alexa Fluor 488 and Propidium Iodide kit (Invitrogen, Waltham, MA, V13241). In brief, EwS cells were washed with PBS and harvested. Cells were then resuspended in Annexin V binding buffer and diluted to approximately 1 × 10^6^ cells. Later, based on the manufacturer’s protocol, the assay was performed in a BD Fusion Aria flow-cytometer and analyzed using FlowJo software, version 10.10.0.

Soft agar colony assay: EwS cells with and without circZNF609-KD were passaged and seeded in a 12-well plate (5000 cells/well) in a soft agar assembly. The bottom agar layer was 0.5% agar mixed with media, and the top layer constituted 0.3% agar mixed with cells and media, and the soft agar assembly was further topped with media to prevent drying of agar. Cells were cultured at 37 °C for 2 weeks. The wells were then stained with 0.01% crystal violet for 30 min and destained with dH20. The colonies were counted under a dissection microscope using a general protocol [24].

Polysome fractionation: Ribosomal subunits and polysomes were resolved from A673 WT, A673 EWS::FlI1, and A673 circZNF609-KD cell extracts by size-exclusion chromatography according to a published protocol [25]. Briefly, cells were grown to 80% confluency before performing lysis. The size-exclusion column (SEC) employed for polysome fractionation was Bio SEC-5, 5 μm particles, 2000 Å, and 7.8 × 300 mm (Agilent Technologies, Santa Clara, CA, USA, 5190-2541), using a Dionex Ultimate 3000 uHPLC system (Thermo Fisher Scientific, Waltham, MA, USA). Later, total RNA was extracted from each of the fractions using Trizol extraction. Equal concentrations of RNA from all the cell lines were used to make cDNA using iScript Reverse Transcription Supermix. The obtained cDNA was used as a template to analyze expression of EWS::FLI1 as well as reference genes (GAPDH and B-Actin) using iTaq Universal SYBR Green Supermix (Bio-RAD, Hercules, CA, USA, 1725124). 

Western blotting: Cells were lysed in cold RIPA buffer containing protease inhibitor cocktail for 30 min. Lysate was centrifuged @ 14,000× *g*/4 °C for 15 min. A total of 10 ug of total protein was boiled with 2× loading dye, and denatured proteins were run on 12% SDS-PAGE gel, following which an overnight wet transfer assembly system was used to transfer the proteins on the gel to a PVDF membrane. The following day, the membrane was washed, blocked, and then treated with the corresponding antibody overnight at 4 °C. The next day, the membrane was washed thrice with 1× TBST and incubated with secondary antibody dissolved in 5% blocking buffer for 1 h at RT. Membrane was later washed with 1× TBST 3–5 times before imaging with SuperSignal™ West Pico PLUS Chemiluminescent Substrate (Thermo Scientific, Waltham, MA, USA, 34580), as per manufacturer’s protocol.

Luciferase assay: Cells were cultured in a 12-well plate at around 40–50% confluency, after which they were transfected with either psiCHECK2-FLI1 3′ UTR F1, psiCHECK2-FLI1 3′ UTR F2, or psiCHECK2-empty vector as a control using Bio-T transfection reagent (Bioland Scientific LLC, Paramount, CA, USA), as per the manufacturer’s protocol. After 24–48 h, lysates were harvested using passive lysis buffer. Later, Firefly and Renilla luciferase readings were taken in a plate reader using the Dual-Luciferase Reagent Assay System (Promega, Madison, WI, USA, E1910), as per the manufacturer’s protocol. Firefly luciferase (expressed constitutively) obtained after transfection was used to normalize bioluminescence values attained for Renilla luciferase, which was expressed in fusion with the 3′ UTR of FLI1. The corrected values were then sequentially normalized to the levels obtained in mock and psiCHECK2-empty vector transfections. Each experiment was repeated in triplicate, and Student’s t-test was performed to obtain *p*-values.

Bioinformatic Prediction and Analyses: Functionally relevant miRNAs targeting FLI1 3′ UTR were predicted using TargetScan and miRDB databases. MiRNAs interacting with circZNF609 and FLI1 3′ UTR were predicted using miRanda v3.3a, circInteractome/TargetScan, and miRDB [26,27,28]. Further comparison of the common miRNAs targeting FLI1 3′ UTR and circZNF609 with miRTarBase v8 identified high-confidence miRNAs potentially involved in the circZNF609/EWS::FLI1/miRNA regulatory axis.

## 3. Results

**Figure 1:**
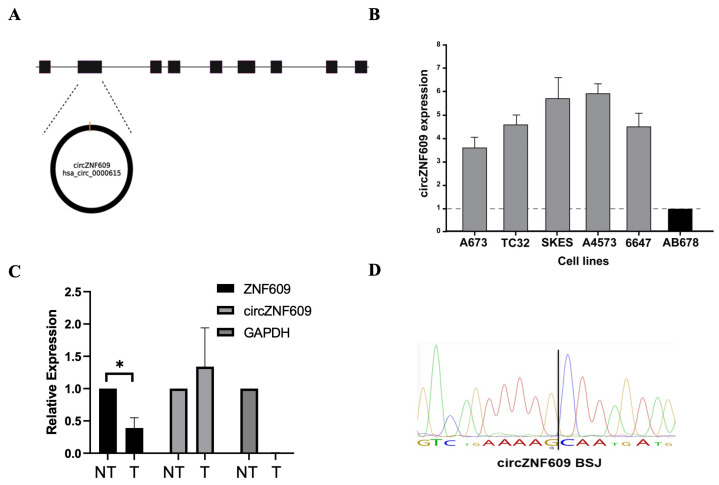
Validating the expression of circZNF609 in EwS cell lines: (**A**). A representative image of the genomic location and back-splicing configuration of circZNF609 (hsa_circ_0000615). (**B**). qRT-PCR data showing circZNF609 expression levels in multiple EwS cell lines as compared to a normal human muscle myoblast cell line (AB678). (**C**). qRT-PCR analysis of A673 cells treated with RNase R demonstrating expression of linear ZNF609, circZNF609, and GAPDH (control), and normalized to mock RNase R treatment. The error bars indicate the standard deviation between triplicates. (**D**). Sanger sequencing of the amplified PCR product of circZNF609 to confirm the unique BSJ. * Indicates a significant difference with a *p*-value < 0.05.

Characterizing CircZNF609 in EwS: CircZNF609 (hsa_circ_0000615) is generated from exon 2 of the ZNF609 pre-mRNA and is 874 nucleotides in length (Figure 1A) [29]. We assessed circZNF609 expression in EwS using RT-qPCR and found it to be constitutively expressed at higher levels in the five different EwS cell lines compared to a control non-Ewing sarcoma cell line (immortalized human myoblast AB678 cells) (Figure 1B). Further validation using divergent primers amplified the circular isoform. Finally, RNase R treatment of total RNA led to a reduction in linear ZNF609 levels while circZNF609 expression persisted, confirming its circular nature; GAPDH was used as a control to demonstrate the effectiveness of RNase R on linear transcripts (Figure 1C). This is expected because RNase R is an exoribonuclease that selectively degrades linear RNA, whereas circRNAs are resistant to RNase R digestion [30,31]. Finally, the PCR product was validated by Sanger sequencing to confirm the presence of the back-splice junction (Figure 1D).

**Figure 2:**
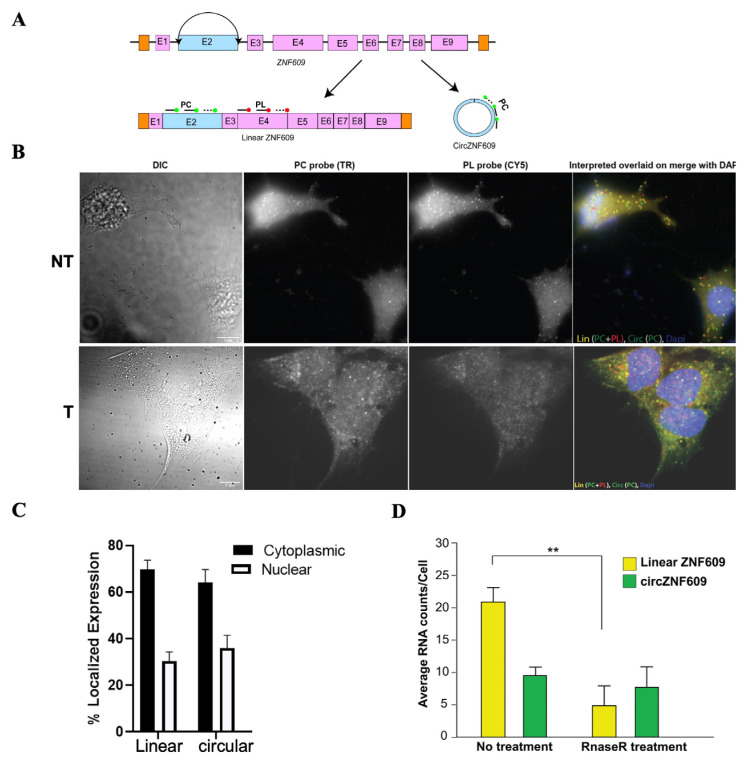
Localization of circZNF609 at single-molecule resolution: (**A**). Schematic depicting simultaneous imaging of linear and circular ZNF609 via circFISH assay. Straight lines terminating in solid circles indicate the specific binding sites of PL (shown as red) and PC (shown as green) probes on their linear and circular ZNF609 targets, respectively. (**B**). Representative image panel of circFISH for ZNF609 in A673 cells with PL and PC probes. From the left: DIC; raw merged z stacks of cells for PL probes labeled with TR; raw merged z stacks of cells for PC probes labeled with Cy5; and a merged image of the two channels with TR spots pseudo-colored red and Cy5 pseudo-colored green, overlaid on DAPI with MATLAB-interpreted spots. Full-length linear, circular, and fragmented linear RNA are represented as yellow, green, and red spots, respectively. The top row shows the non-treated cells (NT), and the bottom row shows the cells after 4 h of RNase R treatment (T). Scale bar is 5 μm. (**C**). Average relative nuclear and cytoplasmic localization of ZNF609 and circZNF609. (**D**). Quantification of linear ZNF609 and circZNF609 signals after MATLAB analysis. The columns represent the average linear ZNF609 and circZNF609 RNA molecules per cell with and without 4 hrs of RNase R treatment, as analyzed in MATLAB. The error bars indicate the 95% confidence interval for at least 100 cells. The error bars indicate the standard deviation between triplicates. ** indicates a significant difference with a *p*-value < 0.005.

Conventional techniques for analyzing non-coding RNAs, including circRNAs, do not provide information about their cellular localization, which is important for understanding their function. We used our recently developed circFISH method [32] to determine the cellular localization of circZNF609 in EwS cell lines at single-molecule resolution (Figure 2A). This method allows simultaneous visualization of both linear and circular isoforms via colocalization analysis of signals from a combination of two probe sets (Figure 2B). Upon quantification, both linear ZNF609 and circZNF609 were found to be predominantly localized in the cytoplasm compared to the nucleus in both A673 and TC32 cells (Figure 2C and Figure S1). The expression levels of circZNF609 were approximately half that of linear ZNF609 (Figure 2D). To confirm whether the signal corresponding to circZNF609 is indeed from the circular isoform and not from any splice variants of linear ZNF609, we performed in situ RNase R treatment [32]. Indeed, we found a significant loss of linear RNA upon treatment with RNaseR (labeled as T; the not-treated panel is labeled as NT), but almost no reduction in circRNA after RNase R treatment, confirming that we are visualizing the circular isoform (Figure 2D). These findings suggest that, being mainly cytoplasmic in localization, circZNF609 may regulate EwS oncogenesis post-transcriptionally, potentially via miRNA or RNA-binding protein (RBP) sponging or via being translated into a functional peptide.

### 3.1. Downregulation of CircZNF609 Impairs EwS Cell Proliferation, Tumorigenicity, and Viability

**Figure 3:**
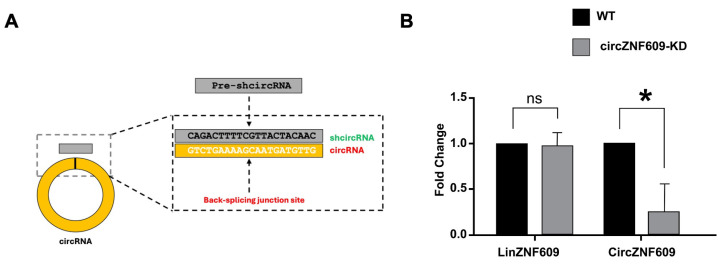
shRNA-mediated circZNF609 knockdown. (**A**). Back-splice junction (BSJ) sequence-specific shRNA designed to knockdown respective circRNA. (**B**). qRT-PCR data showing fold change in the linear ZNF609 and circZNF609 levels in wild-type and circZNF609 knockdown in A673 cells, as normalized to GAPDH levels. The error bars indicate the standard deviation between triplicates. ns Indicates no significant difference. * indicates a significant difference with a *p*-value < 0.05.

To understand the underlying molecular mechanism circZNF609 plays in regulating EwS, we performed a shRNA-mediated knockdown of circZNF609 by targeting the BSJ of circZNF609 (Figure 3A). We found a significant reduction in circZNF609 RNA levels in A673 and TC32 cells, while there was no change in the level of linear ZNF609 expression in either of these cell lines as measured by RT-qPCR (Figure 3B and Figure S2). These results confirmed the specificity and effectiveness of the knockdown.

**Figure 4:**
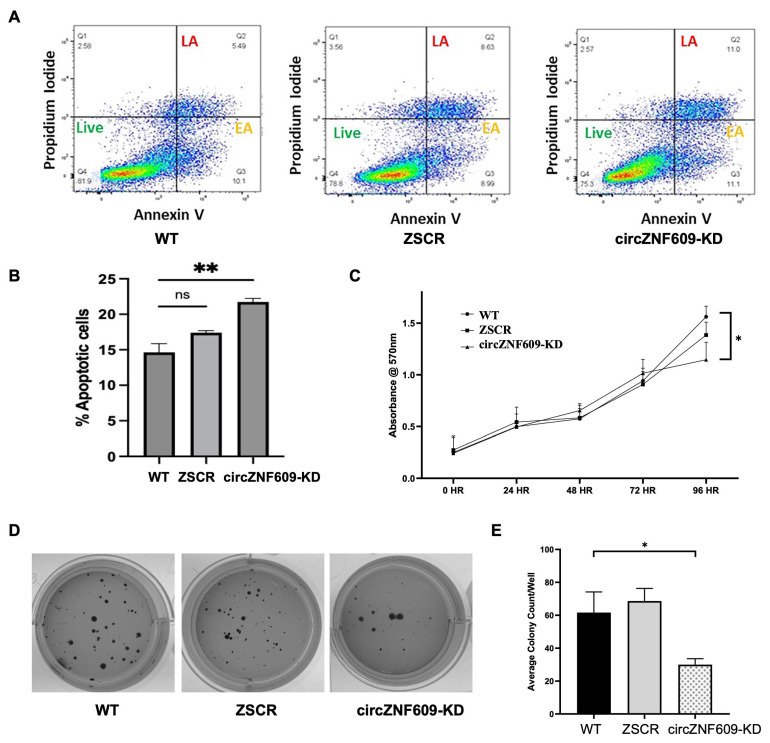
Functional effect of CircZNF609 knockdown in EwS: (**A**). Flow cytometry quadrant analysis distinguishing between healthy and apoptotic (early apoptotic (EA) + late apoptotic (LA)) cells from WT, ZSCR (negative control), and circZNF609-KD cell lines based on their Annexin V and PI levels. Flow cytometry analysis was performed for 20,000 events (cells). (**B**). Quantification of the average percentage of apoptotic cells (EA + LA) from three replicates. (**C**). The average proliferation rate of WT, ZSCR, and circZNF609KD in A673 cells using MTT assay for a period of 4 days with 24 h reading intervals. (**D**). Colonies on soft agar assay analyzed after 14–28 days. (**E**). Quantification of the average colony count per 5000 cells in a 12-well plate for WT, ZSCR, and circZNF609-KD cells after 14 days. The error bars indicate the standard deviation between triplicates. ns Indicates no significant difference. * Indicates a significant difference with a *p*-value < 0.05, and ** indicates a significant difference with a *p*-value <0.005.

To understand if circZNF609 has an oncogenic role in EwS, we evaluated the functional effect of circZNF609 knockdown on the proliferative capacity of EwS cells. Oncogenic circular RNAs are known to result in reduced apoptosis and higher proliferation. We used Annexin V and propidium Iodide (PI) staining to compare cells in early and late apoptosis stages from the WT and circZNF609KD cells. A scrambled KD, ZSCR, was used as a negative control (Figure 4A and Figure S3A). The circZNF609 knockdown in both TC32 and A673 cells resulted in an increase in the number of apoptotic cells as seen in the late apoptotic (LA) and early apoptotic (EA) quadrants for Annexin V and PI double-positive cells, indicating that circZNF609 knockdown induces apoptosis (Figure 4B and Figure S3B). We then analyzed the impact of circZNF609 knockdown on proliferation rate by performing MTT assays and observed a significant reduction in proliferation rate after 72 h in TC32 cells and after 96 h in A673 cells compared to wild-type (WT) cells (Figure 4C and Figure S3C). Furthermore, soft agar colony formation assays showed a significant reduction in the number of colonies formed by circZNF609-KD cells compared to WT cells (Figure 4D,E), indicating reduced tumorigenicity. In summary, these functional assays confirmed that circZNF609 knockdown impairs cell proliferation and tumor growth and induces apoptosis. These results are consistent with existing data reporting that circZNF609 functions as an oncogenic circRNA in other cancers [19,33,34,35]. Our results validated the functional oncogenic role of circZNF609 in EwS for the first time.

### 3.2. Downregulation of CircZNF609 Reduced the Level of EWS::FLI1 Protein in EwS Cells

**Figure 5:**
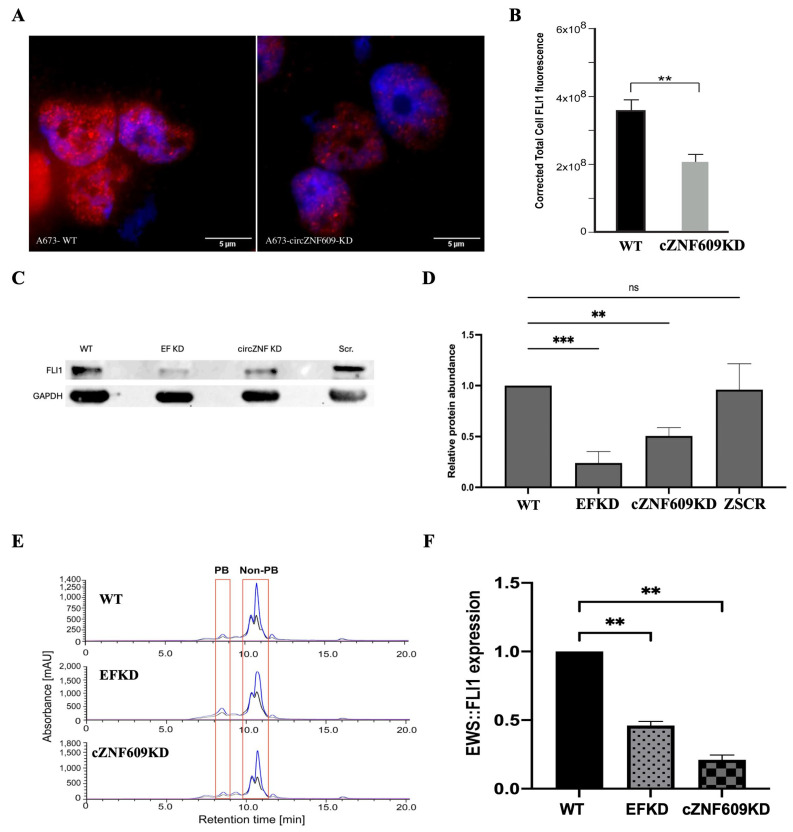
Downregulation of EWS::FLI1 protein levels in circZNF609 KD cells: (**A**). Representative images of A673 (WT and circZNF609-KD) cells after immunofluorescence staining. Raw merged z stacks of cells hybridized with FLI1 primary antibodies tagged with secondary AF647 antibodies (red) overlaid on DAPI (blue). Scale bar is 5 μm. (**B**). Corrected total cell fluorescence (CTCF) of FLI1 protein (representing EWS::FLI1) in WT and circZNF609 KD in A673 cells. The error bars indicate the 95% confidence interval for at least 70 cells. (**C**). Western blot analysis for EWS::FLI1 protein expression across A673 (WT, EFKD, circZNF609-KD, and ZSCR) cells using anti-FLI1 antibody and GAPDH as loading control. (**D**). Quantification of EWS::FLI1 protein expression levels from Western blot across A673 cells (WT, EFKD, circZNF609-KD, and ZSCR). (**E**). The polysome fractionation profile from size-exclusion chromatography (SEC) with the polysome-bound fraction labeled as PB, which is separately collected from non-polysome fractions that include 80S, 60S, 40S, and others [25]. (**F**). qRT-PCR data showing the EWS::FLI1 expression in the polysome fraction obtained using size-exclusion chromatography (SEC) of A673 cells (WT, EF-KD, and circZNF609-KD). Error bars indicate the standard deviation between triplicates. ns Indicates no significant difference. ** Indicates a significant difference with a *p*-value < 0.005. *** Indicates a significant difference with a *p*-value < 0.0001.

To investigate the mechanism by which circZNF609 exerts its functional effects, we examined whether it influences the levels of the oncogenic driver fusion protein EWS::FLI1. We first assessed EWS::FLI1 mRNA levels and found no significant difference between WT and circZNF609 knockdown cells, suggesting that circZNF609 does not affect EWS::FLI1 transcription (Figure S4). Given that circRNAs often regulate gene expression post-transcriptionally, we next evaluated EWS::FLI1 protein levels and observed a reduction in EWS::FLI1 protein in circZNF609 knockdown cell lines, as determined by both immunofluorescence (Figure 5A,B) and Western blot analysis (Figure 5C,D). Further, we also performed polysome fractionation and found a decreased association of EWS::FLI1 mRNA with polysomes in circZNF609 knockdown cells compared to WT cells (Figure 5E,F). An inducible EWS::FLI1 knockdown (EFKD) in A673 cells was used as a positive control that also showed reduced EWS::FLI1 protein, as expected. To confirm that this is not a cell line-specific phenomenon, we repeated the Western blot and immunofluorescence analysis in an additional EwS cell line, TC32, and found similar results (Supplementary Figure S5). These findings further indicate that circZNF609 affects the translation of EWS::FLI1 mRNA.

**Figure 6:**
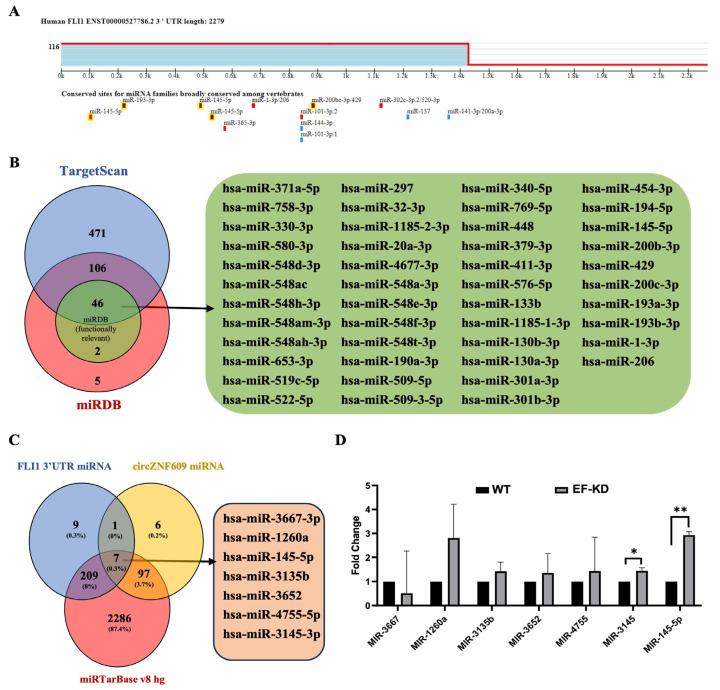
miRNAs targeting EWS::FLI1 and sponged by circZNF609. (**A**). Conserved miRNA binding sites prediction for FLI1 3′ UTR using TargetScan. (**B**). Common miRNAs predicted to bind FLI1 3′ UTR from TargetScan and miRDB. Functionally relevant miRNAs are denoted in green. List of all the functionally relevant miRNAs predicted to target FLI1 3′ UTR from TargetScan and miRDB. (**C**). Common miRNAs predicted to bind FLI1 3′ UTR and circZNF609, and are reported in miRTarBase. (**D**). qRT-PCR data showing fold change in miRNAs in A673 WT and A673 EF-KD cells with common binding elements in FLI1 3′ UTR and circZNF609. The error bars indicate the standard deviation between triplicates. * Indicates a significant difference with a *p*-value < 0.05. ** Indicates a significant difference with a *p*-value < 0.005.

Mechanism of EWS::FLI1 downregulation by circZNF609: Since the most well-studied function of circRNAs is mediated by sponging of miRNAs [11,12,36], we investigated whether a similar regulatory mechanism occurs in EwS cells. Several miRNAs have been reported in the literature to be differentially regulated by EWS::FLI1, although not all directly inhibit EWS::FLI1 expression [37]. Likewise, many miRNAs have been reported to be sponged by circZNF609 in various cancers [18,19,35,38,39,40]. Given the observed reduction in EWS::FLI1 protein in circZNF609 knockdown cells, we aimed to identify miRNAs that both bind to EWS::FLI1 and have binding sites on circZNF609. Using TargetScan and miRDB, we identified 152 miRNAs predicted to bind the 3′ UTR of EWS::FLI1 and found them in both databases (Figure 6A), which we further narrowed down to 46 that are functionally relevant miRNAs using miRDB (Figure 6B). We then identified all the miRNAs predicted to have a binding site for circZNF609 and compared them to all miRNAs predicted to bind FLI1 3′ UTR. Finally, we compared this analysis to the miRTarBase v8 hg to find which of these common miRNAs are reported to be expressed in human cells, and we identified seven common miRNAs (Figure 6C). All seven were tested by qRT-PCR to determine their levels in EwS cells, and we found that miR-145-5p showed the most significant EWS::FLI1-dependent expression (Figure 6D). Numerous studies have reported that circZNF609 acts by sponging miR-145-5p, as seen in glioblastoma [18,39,41]. Furthermore, miR-145-5p is a key miRNA in EwS, and its reduction has been reported to promote oncogenesis in EwS via a yet unknown mechanism [14,42]. Finally, miR145-5p has been shown to regulate the expression of FLI1 in osteosarcoma [43].

**Figure 7:**
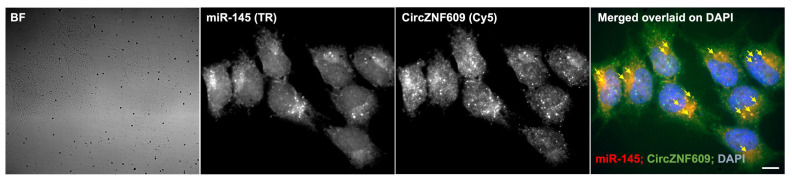
Simultaneous staining of circRNA and miRNA: Representative image panel of A673 cells hybridized with antibodies for miR-145 (TR) and probes for circZNF609 (Cy5). A merged image of the two channels with miR-145 spots pseudo-colored in red and circZNF609 pseudo-colored in green, overlaid on DAPI. Yellow arrows point to the colocalization between miR-145 and circZNF609. Scale bar is 5 μm.

Direct visualization of circZNF609 and miR145-5p interaction: To directly visualize the interaction between circZNF609 and miR-145-5p, we combined our circFISH assay with miRNA imaging to assess their co-localization. CircZNF609 was detected using probes labeled with Cy5, and after hybridization with circFISH probes, cells were further stained with miRNA probes requiring signal amplification. We observed that a subset of circZNF609 RNA molecules co-localized with the miR-145-5p signal. Although the signals were somewhat diminished due to multiple rounds of washing, staining, and signal processing, the data clearly demonstrate that circZNF609 has a binding site for miR-145-5p and that this interaction occurs in A673 cells (Figure 7).

**Figure 8:**
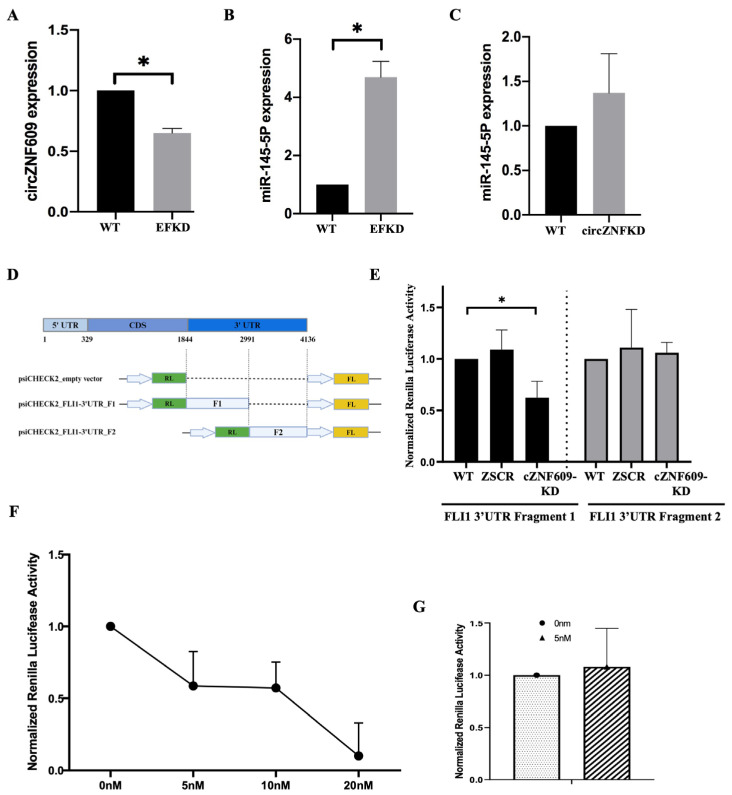
Function validation of circZNF609 regulating miR145 function in EwS: (**A**). qRT-PCR to quantify the expression of circZNF609 in WT and EFKD cells. (**B**). Expression of miR145-5p in WT and EFKD. (**C**). Expression of miR145-5p in WT and circZNF609-KD cells. (**D**). Schematic of luciferase constructs expressing fragments of FLI1 3′ UTR. (**E**). Renilla luciferase activity normalized to empty vector in WT, ZSCR (negative control), and circZNF609-KD cells for both fragments 1 and 2 of FLI1 3′ UTR. (**F**). Dose response curve of luciferase activity normalized to empty vector upon addition of a miR145 mimic in WT cells expressing fragment 1 of FLI1 3′ UTR. (**G**). Luciferase activity normalized to empty vector upon addition of a negative control miRNA mimic in TC32 WT cells expressing fragment 1 of FLI1 3′ UTR. The error bars indicate the standard deviation between triplicates. * Indicates a significant difference with a *p*-value < 0.05.

Functional validation of the role of circZNF609 sponging of miR145-5p in EwS: It is well established that miR-145-5p is significantly repressed by EWS::FLI1 and functions in a feedback regulatory loop in EwS, although the mechanism of miR-145-5p suppression is not understood [14,44,45]. Interestingly, we observed a reduction in circZNF609 levels in EWS::FLI1 knockdown (EFKD) cells, indicating that its expression is positively correlated with EWS::FLI1 (Figure 8A). Consistent with the existing literature, miR-145-5p levels showed a significant increase in EFKD cells (Figure 8B), but there was no reduction in miR-145-5p levels in circZNF609 knockdown cells, suggesting that circZNF609 does not regulate the expression of miR-145-5p (Figure 8C) [14]. This is not unexpected, as circRNAs typically do not affect the expression but rather the function of their target miRNAs [46]. To assess the impact of circZNF609 on miR-145-5p function, we performed a luciferase assay by cloning two fragments of the FLI1 3′ UTR into the psiCHECK2 vector downstream of the Renilla luciferase coding region. Fragment 1 contains the binding site for miR-145-5p, while fragment 2 does not (Figure 8D). TC32 cells (WT, ZSCR, and circZNF609-KD) were transfected with these constructs, along with empty vector controls. We observed a change in luciferase expression only with FLI1 3′ UTR fragment 1, with reduced luciferase activity in circZNF609-KD cells, while fragment 2 remained unchanged (Figure 8E), suggesting that circZNF609 sponges miR-145-5p. Finally, to show that the effect is due to the sponging of miR-145 by circZNF609, we added a mimic of miR145-5p to WT cells that should overexpress the miRNA and thus reverse the sponging effect of circZNF609. Indeed, addition of a miR-145-5p mimic showed a dose-dependent response reduction in luciferase activity in TC32 WT cells expressing fragment 1 of FLI1 3′ UTR (Figure 8F), with a marked effect seen at 5 nM concentration of the mimic. To further confirm the specificity of this effect, we repeated this experiment using 5 nM of a negative control mimic, which did not affect luciferase activity (Figure 8G). These results confirmed that the effect is specific to miR-145 binding to the 3′ UTR, which is also a target of circZNF609. Together, the direct visualization of the circRNA:miRNA interaction and the functional luciferase assay validated and provided the first evidence for the functional circZNF609–miR-145–EWS::FLI1 regulatory axis in EwS.

circZNF609 functions as a regulatory RNA in EwS: Although circRNAs are generally classified as non-coding RNAs, a few have been shown to be translated into short peptides via cap-independent mechanisms, such as internal ribosome entry sites [47]. Notably, circZNF609 is among the few circRNAs with demonstrated coding potential, and its peptide has been shown to have functional roles in myogenesis and acute kidney disease [29,48]. This prompted us to investigate whether the peptide form of circZNF609 plays a role in EwS. We performed polysome fractionation and found no significant changes in the association of circZNF609 RNA with polysome fractions among WT, EFKD, and circZNF609KD cells, suggesting that if the peptide is produced, it does not play a functional role in EwS. These results confirm that circZNF609 functions as a regulatory RNA in EwS (Supplementary Figure S6).

## 4. Discussion

Over the past decade, ample evidence has demonstrated the impact of circRNAs on the development and progression of various diseases, particularly human cancers such as lung, prostate, colon, breast, and hepatocellular carcinomas, as well as osteosarcoma [13,19,35,49,50,51,52,53]. CircRNAs are highly stable due to their resistance to exonucleases and have been detected in extracellular vesicles and bodily fluids, making them promising candidates for biomarkers and therapeutics [9,13,54,55].

**Figure 9:**
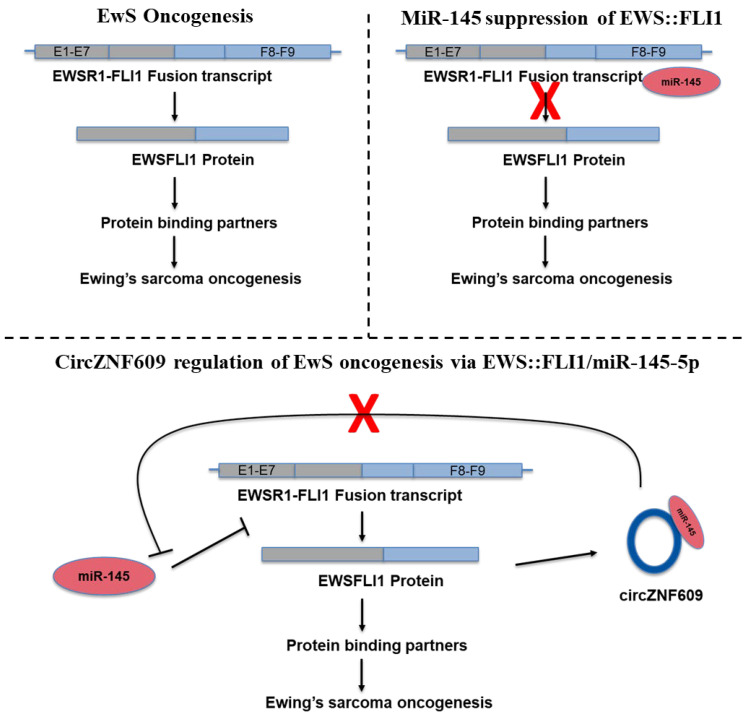
EWS::FLI1 recruits circZNF609 to repress miR145–5p function: Proposed model for EWS::FLI1 regulation by circZNF609 in EwS showing under normal conditions; miR145-5p degrades the EWS::FLI1 mRNA that, upon expression of EWS::FLI1 protein, circZNF609 is upregulated, which sponges the miR145–5p and de-represses its effect, and thus, high amounts of EWS::FLI1 are achieved in the cell, promoting oncogenesis in EwS. The pointed arrows indicate activation/direction of the process, blunt end arrows indicate inhibition, A red “X” means a blocked step in the pathway.

CircZNF609 is a very well-studied oncogenic circRNA [35]. Existing research has demonstrated the importance of circZNF609 in regulating cell proliferation, migration, and metastasis in several human malignancies, including hepatocellular carcinoma, breast cancer, lung adenocarcinoma, gastric cancer, and glioma [17,18,35,56,57]. However, the expression and function of circZNF609 have not previously been reported in EwS. Here, we present the first characterization of a functional circular RNA in EwS. We validated the expression, role, and mechanism of action of circZNF609 in EwS and identified the circZNF609:miR-145-5p:FLI1 regulatory axis. Our data show that circZNF609 is expressed in an EWS::FLI1-dependent manner, and it is well established that miR-145-5p is a critical miRNA capable of suppressing EWS::FLI1 expression. Furthermore, FLI1 is not expressed in normal cells, which is why EWS::FLI1 is often detected and studied using antibodies against the 3′ end of FLI1 [58]. Integrating our new functional data with existing studies, we propose a model in which, under normal conditions, cells express miR-145-5p, which binds to the 3′ UTR of FLI1 and keeps EWS::FLI1 translationally inhibited after the translocation occurs. However, as EWS::FLI1 expression increases, its concentration likely surpasses the regulatory capacity of miR-145-5p, allowing EWS::FLI1 protein to accumulate. Early expression of EWS::FLI1 induces circZNF609 and other effectors that repress miR-145-5p expression by yet unknown mechanisms. The expression of circZNF609 leads to sponging of miR-145-5p, thereby blocking its function and enabling higher levels of EWS::FLI1 protein, which, in turn, promotes aberrant gene expression and increases proliferation, invasiveness, and growth in EwS (Figure 9). This positive feedback loop ensures that EWS::FLI1 protein concentrations remain high in the cells, representing a novel strategy by which EWS::FLI1 evades repression by miRNAs.

This is the first report describing the role of circular RNAs in EwS. A recent work by Chen et al. has reported another circRNA called circCAMSAP1 to sponge miR-145 to de-repress FLI1 expression in osteosarcoma in both cell lines and animal models, providing a comparative perspective on circRNA functions across different sarcomas as well as the biological relevance of reducing miR145-FLI1 interaction [43]; however, this circRNA has not been identified in EwS cell lines. Through genome profiling, we identified more circRNAs, of which several, including circZNF609, are upregulated, and some are downregulated in EwS, indicating diverse functional roles for these regulatory RNAs that remain to be explored (unpublished data). We acknowledge that a limitation of this study is the lack of analysis of circZNF609 levels in patient samples. Future studies are planned to test the expression of circZNF609 in patient samples, as well as to functionally characterize the role of other circRNAs. A recent study showed that EWS::FLI1 is not just a “rogue” transcription factor (regulating gene expression at the DNA level), but it also functions independently as an mRNA decay factor (regulating the lifespan of genetic messages) [59]. By identifying and characterizing the mechanism of action of the first circular RNA in EwS, our study provides another possible function of EWS::FLI1 as an “miRNA silencer” and will set the foundation for future research aimed at developing better diagnostic markers and therapeutic targets for EwS.

## Data Availability

The original contributions presented in this study are included in the article/Supplementary Material. Further inquiries can be directed to the corresponding author.

## Supplementary Materials

The following supporting information can be downloaded at https://www.mdpi.com/article/10.3390/biomedicines14010129/s1: Figure S1: Visualization of circZNF609 in additional EwS cell line; Figure S2: shRNA mediated circZNF609 knockdown in additional EwS cell line; Figure S3: Functional assays demonstrating oncogenic role of circZNF609 in additional EwS cell line; Figure S4: EWS::FLI1 mRNA levels unaltered by circZNF609 knockdown; Figure S5: Downregulation of EWS::FLI1 protein levels in circZNF609 KD cells; Figure S6: CircZNF609 associated peptide expression in EwS cells; Table S1: shRNA Sequences for circZNF60 Knockdown; Table S2: Oligo Sequences for ZNF609 PC and PL CircFISH Probes Sets; Table S3: List of Primers used; Table S4: List of antibodies used.

## References

[1] Grünewald T.G.P., Cidre-Aranaz F., Surdez D., Tomazou E.M., de Álava E., Kovar H., Sorensen P.H., Delattre O., Dirksen U. (2018). Ewing Sarcoma. Nat. Rev. Dis. Primers.

[2] May W.A., Gishizky M.L., Lessnick S.L., Lunsford L.B., Lewis B.C., Delattre O., Zucman J., Thomas G., Denny C.T. (1993). Ewing Sarcoma 11; 22 Translocation Produces a Chimeric Transcription Factor That Requires the DNA-Binding Domain Encoded by FLI1 for Transformation. Proc. Natl. Acad. Sci. USA.

[3] May W.A., Lessnick S.L., Braun B.S., Klemsz M., Lewis B.C., Lunsford L.B., Hromas R., Denny C.T. (1993). The Ewing’s Sarcoma EWS/FLI-1 Fusion Gene Encodes a More Potent Transcriptional Activator and Is a More Powerful Transforming Gene than FLI-1. Mol. Cell. Biol..

[4] Figuerola-Bou E., Ríos-Astorch C., Blanco E., Sánchez-Jiménez M., Táboas P., Fernández G., Gómez-González S., Muñoz O., Castellano-Escuder P., Perez-Jaume S. (2025). KDM6 Demethylases Contribute to EWSR1::FLI1-Driven Oncogenic Reprogramming in Ewing Sarcoma. Cancer Res..

[5] Fan Z., Dong S., Wang N., Khawar M.B., Wang J., Sun H. (2024). Unlocking Epigenetics for Precision Treatment of Ewing’s Sarcoma. Chin. J. Cancer Res..

[6] Deng Q., Natesan R., Cidre-Aranaz F., Arif S., Liu Y., Rasool R.U., Wang P., Mitchell-Velasquez E., Das C.K., Vinca E. (2022). Oncofusion-Driven de Novo Enhancer Assembly Promotes Malignancy in Ewing Sarcoma via Aberrant Expression of the Stereociliary Protein LOXHD1. Cell Rep..

[7] Derrien T., Johnson R., Bussotti G., Tanzer A., Djebali S., Tilgner H., Guernec G., Martin D., Merkel A., Knowles D.G. (2012). The GENCODE v7 Catalog of Human Long Noncoding RNAs: Analysis of Their Gene Structure, Evolution, and Expression. Genome Res..

[8] Kristensen L.S., Andersen M.S., Stagsted L.V.W., Ebbesen K.K., Hansen T.B., Kjems J. (2019). The Biogenesis, Biology and Characterization of Circular RNAs. Nat. Rev. Genet..

[9] Li Y., Zeng X., He J., Gui Y., Zhao S., Chen H., Sun Q., Jia N., Yuan H. (2018). Circular RNA as a Biomarker for Cancer: A Systematic Meta-Analysis. Oncol. Lett..

[10] Jeck W.R., Sorrentino J.A., Wang K., Slevin M.K., Burd C.E., Liu J., Marzluff W.F., Sharpless N.E. (2013). Circular RNAs Are Abundant, Conserved, and Associated with ALU Repeats. RNA.

[11] Memczak S., Jens M., Elefsinioti A., Torti F., Krueger J., Rybak A., Maier L., Mackowiak S.D., Gregersen L.H., Munschauer M. (2013). Circular RNAs Are a Large Class of Animal RNAs with Regulatory Potency. Nature.

[12] Hansen T.B., Jensen T.I., Clausen B.H., Bramsen J.B., Finsen B., Damgaard C.K., Kjems J. (2013). Natural RNA Circles Function as Efficient microRNA Sponges. Nature.

[13] Su M., Xiao Y., Ma J., Tang Y., Tian B., Zhang Y., Li X., Wu Z., Yang D., Zhou Y. (2019). Circular RNAs in Cancer: Emerging Functions in Hallmarks, Stemness, Resistance and Roles as Potential Biomarkers. Mol. Cancer.

[14] Ban J., Jug G., Mestdagh P., Schwentner R., Kauer M., Aryee D.N.T., Schaefer K.-L., Nakatani F., Scotlandi K., Reiter M. (2011). Hsa-Mir-145 Is the Top EWS-FLI1-Repressed microRNA Involved in a Positive Feedback Loop in Ewing’s Sarcoma. Oncogene.

[15] Barrett C., Budhiraja A., Parashar V., Batish M. (2021). The Landscape of Regulatory Noncoding RNAs in Ewing’s Sarcoma. Biomedicines.

[16] McKinsey E.L., Parrish J.K., Irwin A.E., Niemeyer B.F., Kern H.B., Birks D.K., Jedlicka P. (2011). A Novel Oncogenic Mechanism in Ewing Sarcoma Involving IGF Pathway Targeting by EWS/Fli1-Regulated microRNAs. Oncogene.

[17] He Y., Huang H., Jin L., Zhang F., Zeng M., Wei L., Tang S., Chen D., Wang W. (2020). CircZNF609 Enhances Hepatocellular Carcinoma Cell Proliferation, Metastasis, and Stemness by Activating the Hedgehog Pathway through the Regulation of miR-15a-5p/15b-5p and GLI2 Expressions. Cell Death Dis..

[18] Wang S., Xue X., Wang R., Li X., Li Q., Wang Y., Xie P., Kang Y., Meng R., Feng X. (2018). CircZNF609 Promotes Breast Cancer Cell Growth, Migration, and Invasion by Elevating p70S6K1 via Sponging miR-145-5p. Cancer Manag. Res..

[19] Qian Y., Li Y., Li R., Yang T., Jia R., Ge Y.-Z. (2021). Circ-ZNF609: A Potent circRNA in Human Cancers. J. Cell. Mol. Med..

[20] Batish M., Tyagi S. (2019). Fluorescence In Situ Imaging of Dendritic RNAs at Single-Molecule Resolution. Curr. Protoc. Neurosci..

[21] Romero B., Hoque P., Robinson K.G., Lee S.K., Sinha T., Panda A., Shrader M.W., Parashar V., Akins R.E., Batish M. (2024). The Circular RNA circNFIX Regulates MEF2C Expression in Muscle Satellite Cells in Spastic Cerebral Palsy. J. Biol. Chem..

[22] Schneider C.A., Rasband W.S., Eliceiri K.W. (2012). NIH Image to ImageJ: 25 Years of Image Analysis. Nat. Methods.

[23] McCloy R.A., Rogers S., Caldon C.E., Lorca T., Castro A., Burgess A. (2014). Partial Inhibition of Cdk1 in G 2 Phase Overrides the SAC and Decouples Mitotic Events. Cell Cycle.

[24] Du F., Zhao X., Fan D. (2017). Soft Agar Colony Formation Assay as a Hallmark of Carcinogenesis. Bio Protoc..

[25] Yoshikawa H., Larance M., Harney D.J., Sundaramoorthy R., Ly T., Owen-Hughes T., Lamond A.I. (2018). Efficient Analysis of Mammalian Polysomes in Cells and Tissues Using Ribo Mega-SEC. eLife.

[26] Chen Y., Wang X. (2020). miRDB: An Online Database for Prediction of Functional microRNA Targets. Nucleic Acids Res..

[27] Dudekula D.B., Panda A.C., Grammatikakis I., De S., Abdelmohsen K., Gorospe M. (2016). CircInteractome: A Web Tool for Exploring Circular RNAs and Their Interacting Proteins and microRNAs. RNA Biol..

[28] McGeary S.E., Lin K.S., Shi C.Y., Pham T.M., Bisaria N., Kelley G.M., Bartel D.P. (2019). The Biochemical Basis of microRNA Targeting Efficacy. Science.

[29] Legnini I., Di Timoteo G., Rossi F., Morlando M., Briganti F., Sthandier O., Fatica A., Santini T., Andronache A., Wade M. (2017). Circ-ZNF609 Is a Circular RNA That Can Be Translated and Functions in Myogenesis. Mol. Cell.

[30] Suzuki H., Zuo Y., Wang J., Zhang M.Q., Malhotra A., Mayeda A. (2006). Characterization of RNase R-Digested Cellular RNA Source That Consists of Lariat and Circular RNAs from Pre-mRNA Splicing. Nucleic Acids Res..

[31] Vincent H.A., Deutscher M.P. (2006). Substrate Recognition and Catalysis by the Exoribonuclease RNase R. J. Biol. Chem..

[32] Koppula A., Abdelgawad A., Guarnerio J., Batish M., Parashar V. (2022). CircFISH: A Novel Method for the Simultaneous Imaging of Linear and Circular RNAs. Cancers.

[33] Rossi F., Legnini I., Megiorni F., Colantoni A., Santini T., Morlando M., Di Timoteo G., Dattilo D., Dominici C., Bozzoni I. (2019). Circ-ZNF609 Regulates G1-S Progression in Rhabdomyosarcoma. Oncogene.

[34] Wang J., Lin Y., Jiang D.-H., Yang X., He X.-G. (2021). CircRNA ZNF609 Promotes Angiogenesis in Nasopharyngeal Carcinoma by Regulating miR-145/STMN1 Axis. Kaohsiung J. Med. Sci..

[35] Wang S., Wu J., Wang Z., Gong Z., Liu Y., Wang Z. (2022). Emerging Roles of Circ-ZNF609 in Multiple Human Diseases. Front. Genet..

[36] Panda A.C. (2018). Circular RNAs Act as miRNA Sponges. Adv. Exp. Med. Biol..

[37] Mosakhani N., Guled M., Leen G., Calabuig-Fariñas S., Niini T., Machado I., Savola S., Scotlandi K., López-Guerrero J.A., Llombart-Bosch A. (2012). An Integrated Analysis of miRNA and Gene Copy Numbers in Xenografts of Ewing’s Sarcoma. J. Exp. Clin. Cancer Res..

[38] Du S., Li H., Lu F., Zhang S., Tang J. (2021). Circular RNA ZNF609 Promotes the Malignant Progression of Glioma by Regulating miR-1224-3p/PLK1 Signaling. J. Cancer.

[39] Ghadami E., Gorji A., Pour-Rashidi A., Noorbakhsh F., Kabuli M., Razipour M., Choobineh H., Maghsudlu M., Damavandi E., Ghadami M. (2024). CircZNF609 and circNFIX as Possible Regulators of Glioblastoma Pathogenesis via miR-145-5p/EGFR Axis. Sci. Rep..

[40] Ren L., Huo X., Zhao Y. (2024). CircZNF609/miR-324-5p/Voltage-Dependent Anion Channel 1 Axis Promotes Malignant Progression of Ovarian Cancer Cells. iScience.

[41] Liu Z., Pan H.-M., Xin L., Zhang Y., Zhang W.-M., Cao P., Xu H.-W. (2019). Circ-ZNF609 Promotes Carcinogenesis of Gastric Cancer Cells by Inhibiting miRNA-145-5p Expression. Eur. Rev. Med. Pharmacol. Sci..

[42] Guzel Tanoglu E., Ozturk S. (2021). miR-145 Suppresses Epithelial-Mesenchymal Transition by Targeting Stem Cells in Ewing Sarcoma Cells. Bratisl. Lekárske Listy.

[43] Chen Z., Xu W., Zhang D., Chu J., Shen S., Ma Y., Wang Q., Liu G., Yao T., Huang Y. (2021). circCAMSAP1 Promotes Osteosarcoma Progression and Metastasis by Sponging miR-145-5p and Regulating FLI1 Expression. Mol. Ther. Nucleic Acids.

[44] Cui S.-Y., Wang R., Chen L.-B. (2014). MicroRNA-145: A Potent Tumour Suppressor That Regulates Multiple Cellular Pathways. J. Cell. Mol. Med..

[45] Fan L., Wu Q., Xing X., Wei Y., Shao Z. (2012). MicroRNA-145 Targets Vascular Endothelial Growth Factor and Inhibits Invasion and Metastasis of Osteosarcoma Cells. Acta Biochim. Biophys. Sin..

[46] Silenzi V., D’Ambra E., Santini T., D’Uva S., Setti A., Salvi N., Nicoletti C., Scarfò R., Cordella F., Mongiardi B. (2024). A Tripartite circRNA/mRNA/miRNA Interaction Regulates Glutamatergic Signaling in the Mouse Brain. Cell Rep..

[47] Hwang H.J., Kim Y.K. (2024). Molecular Mechanisms of Circular RNA Translation. Exp. Mol. Med..

[48] Ouyang X., He Z., Fang H., Zhang H., Yin Q., Hu L., Gao F., Yin H., Hao T., Hou Y. (2023). A Protein Encoded by Circular ZNF609 RNA Induces Acute Kidney Injury by Activating the AKT/mTOR-Autophagy Pathway. Mol. Ther..

[49] D’Ambra E., Capauto D., Morlando M. (2019). Exploring the Regulatory Role of Circular RNAs in Neurodegenerative Disorders. Int. J. Mol. Sci..

[50] Guo L., Jia L., Luo L., Xu X., Xiang Y., Ren Y., Ren D., Shen L., Liang T. (2022). Critical Roles of Circular RNA in Tumor Metastasis via Acting as a Sponge of miRNA/isomiR. Int. J. Mol. Sci..

[51] Soghli N., Qujeq D., Yousefi T., Soghli N. (2020). The Regulatory Functions of Circular RNAs in Osteosarcoma. Genomics.

[52] Yang X., Mei J., Wang H., Gu D., Ding J., Liu C. (2020). The Emerging Roles of Circular RNAs in Ovarian Cancer. Cancer Cell Int..

[53] Zhang Z., Wang C., Zhang Y., Yu S., Zhao G., Xu J. (2020). CircDUSP16 Promotes the Tumorigenesis and Invasion of Gastric Cancer by Sponging miR-145-5p. Gastric Cancer.

[54] Abdelgawad A., Huang Y., Gololobova O., Yu Y., Witwer K.W., Parashar V., Batish M. (2025). Defining the Parameters for Sorting of RNA Cargo Into Extracellular Vesicles. J. Extracell. Vesicles.

[55] Hoque P., Romero B., Akins R.E., Batish M. (2023). Exploring the Multifaceted Biologically Relevant Roles of circRNAs: From Regulation, Translation to Biomarkers. Cells.

[56] Liu S., Yang N., Jiang X., Wang J., Dong J., Gao Y. (2021). FUS-Induced Circular RNA ZNF609 Promotes Tumorigenesis and Progression via Sponging miR-142-3p in Lung Cancer. J. Cell. Physiol..

[57] Wu L., Xia J., Yang J., Shi Y., Xia H., Xiang X., Yu X. (2018). Circ-ZNF609 Promotes Migration of Colorectal Cancer by Inhibiting Gli1 Expression via microRNA-150. J. BUON.

[58] Melot T., Gruel N., Doubeikovski A., Sevenet N., Teillaud J.L., Delattre O. (1997). Production and Characterization of Mouse Monoclonal Antibodies to Wild-Type and Oncogenic FLI-1 Proteins. Hybridoma.

[59] Galvan B., Ongena L., Bruyr J., Fettweis G., Lucarelli E., Lavergne A., Mariavelle E., O’Grady T.M., Hassoun Z.E.O., Claes M. (2025). Subversion of mRNA Degradation Pathways by EWSR1::FLI1 Represents a Therapeutic Vulnerability in Ewing Sarcoma. Nat. Commun..

