# Supplementary material for: Circuitous Ways of EWS::FLI1 Using Circular RNA ZNF609 to Evade Translational Repression by miR-145 in Ewing’s Sarcoma
Source: Biomedicines. 2026 Jan 8;14(1):129. doi: 10.3390/biomedicines14010129 (PMC12838600; doi:10.3390/biomedicines14010129)
Supplement: Supplementary file 1 [file biomedicines-14-00129-s001.zip › biomedicines-4037674-supplementary.pdf]

Figure S1

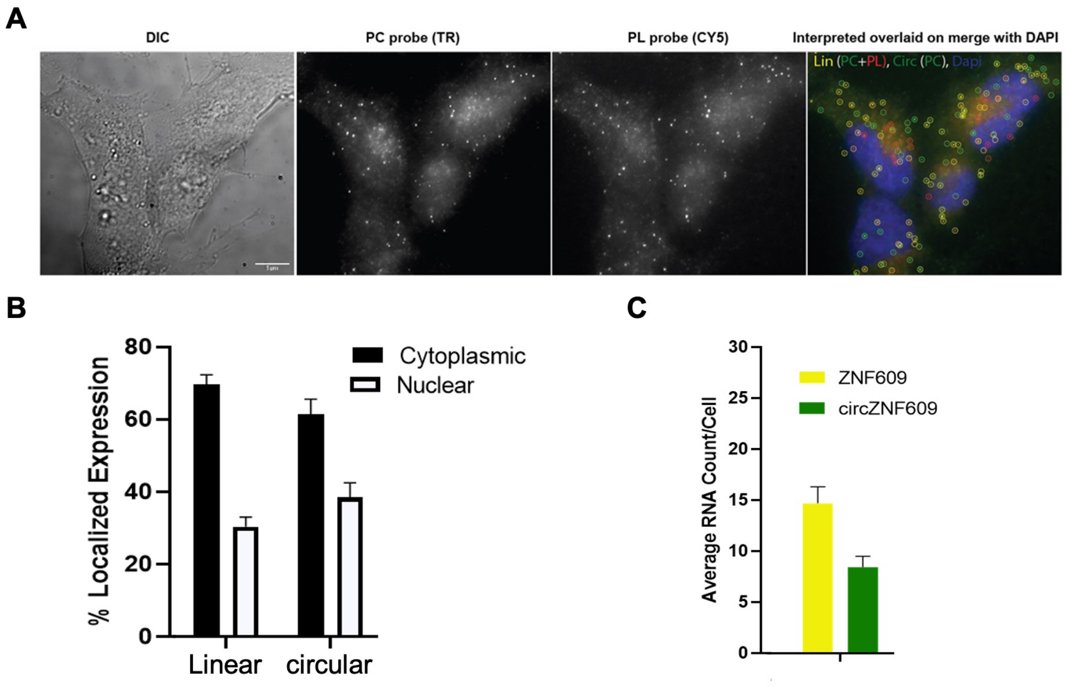

Figure S1: Visualization of circZNF609 in additional EwS cell line: TC32 cells were hybridized with PC and PL probe sets as described in figure 1. Representative image panel of circFISH for ZNF609 in A673 cells with the PL and PC probes. From the left: DIC; raw merged z stacks of cells for PL probes labeled with TR; raw merged z stacks of cells for PC probes labeled with Cy5; and a merged image of the two channels with TR spots pseudo-colored red and Cy5 pseudo-colored green, overlaid on DAPI with MATLAB-interpreted spots. Full-length linear, circular and fragmented linear RNA is represented as yellow, green, and red spots, respectively. The scale bar is 5 µm. B. Average relative nuclear and cytoplasmic localization of ZNF609 and circZNF609. C. Quantification of linear ZNF609 and circZNF609 signals after MATLAB analysis. The columns represent the average linear ZNF609 and circZNF609 RNA molecules per cell. The error bars indicate the 95% confidence interval for at least 100 cells. The error bars indicate the standard deviation between triplicates.

Figure S2

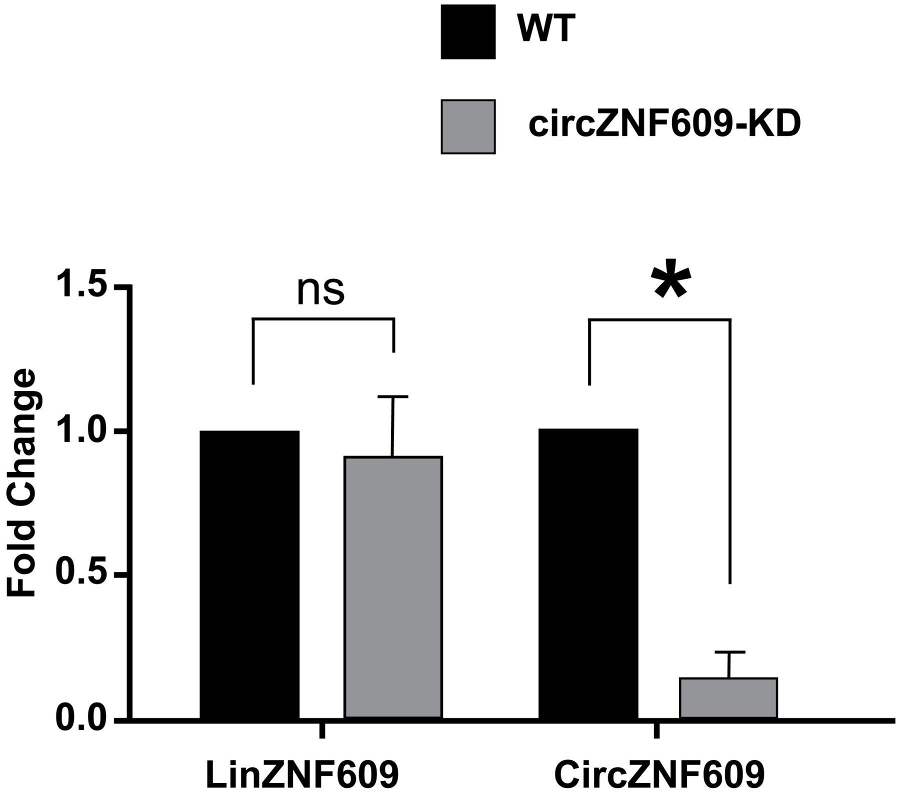

Figure S2: **shRNA mediated circZNF609 knockdown in additional EwS cell line.** qRT-PCR data showing fold change in the linear ZNF609 and circZNF609 levels in wildtype and circZNF609 knockdown TC32 cells as normalized to GAPDH levels. The error bars indicate the standard deviation between triplicates. * Indicates a significant difference with a p-value < 0.05.

Figure S3

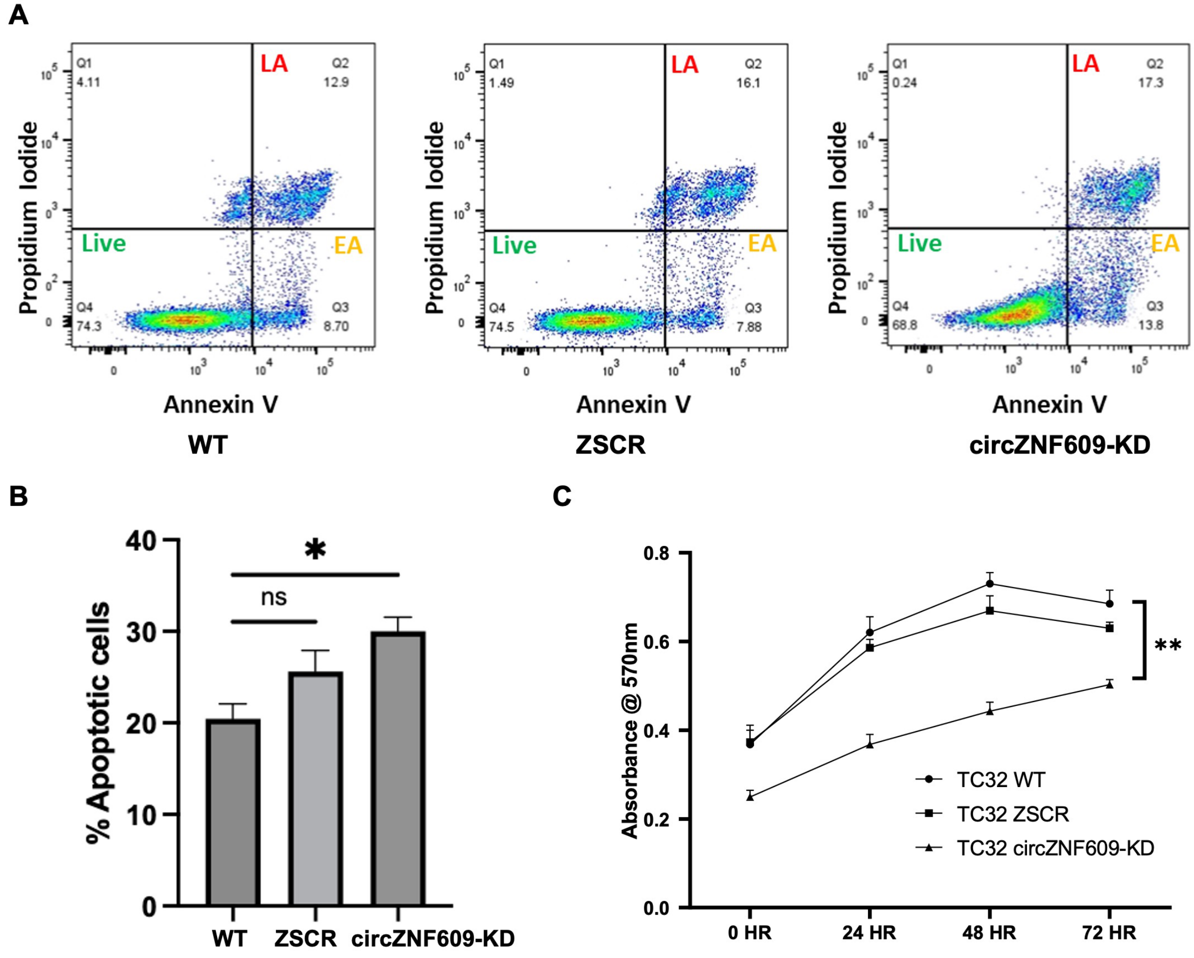

Figure S3: Functional assays demonstrating oncogenic role of circZNF609 in additional EwS cell line: A Flow cytometry quadrant analysis distinguishing between healthy and apoptotic (Early apoptotic (EA) + Late apoptotic (LA) cells from WT, ZSCR (negative control) and circZNF609-KD TC32 cell lines based on their Annexin V and PI levels. Flow cytometry analysis was performed for 20,000 events (cells). **B.** Bar graph showing quantification showing average percent apoptotic cells (EA +LA) from three replicates. C. The average proliferation rate of WT, ZSCR and circZNF609KD in TC32 cells using MTT assay for a period of 3 days with 24 hour reading intervals. The error bars indicate the standard deviation between triplicates. * Indicates a significant difference with a p-value < 0.05, ** indicates a significant difference with a p-value <0.001.

Figure S4

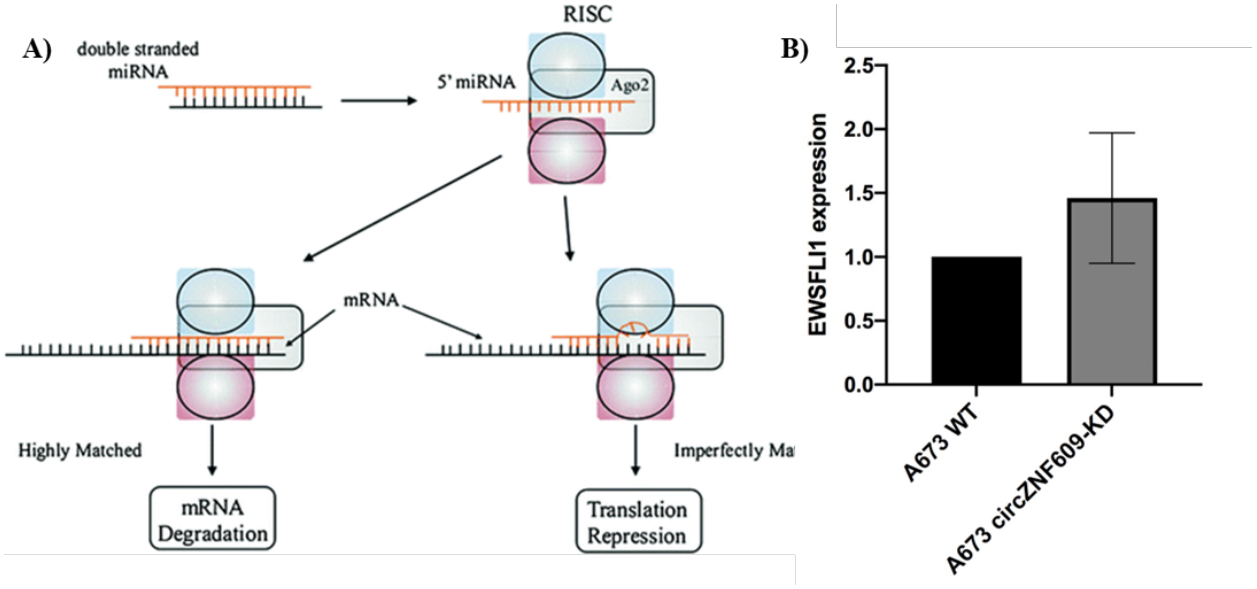

Figure S4: EWS::FLI1 mRNA levels unaltered by circZNF609 knockdown. A) miRNA association to the target mRNA(perfectly matched or imperfectly matched) determines if the mRNA is degraded or undergoes translational repression. B) qRT-PCR data showing EWS::FLI1 mRNA levels between A673 WT and circZNF609-KD cells. The error bars indicate the standard deviation between triplicates.

Figure S5

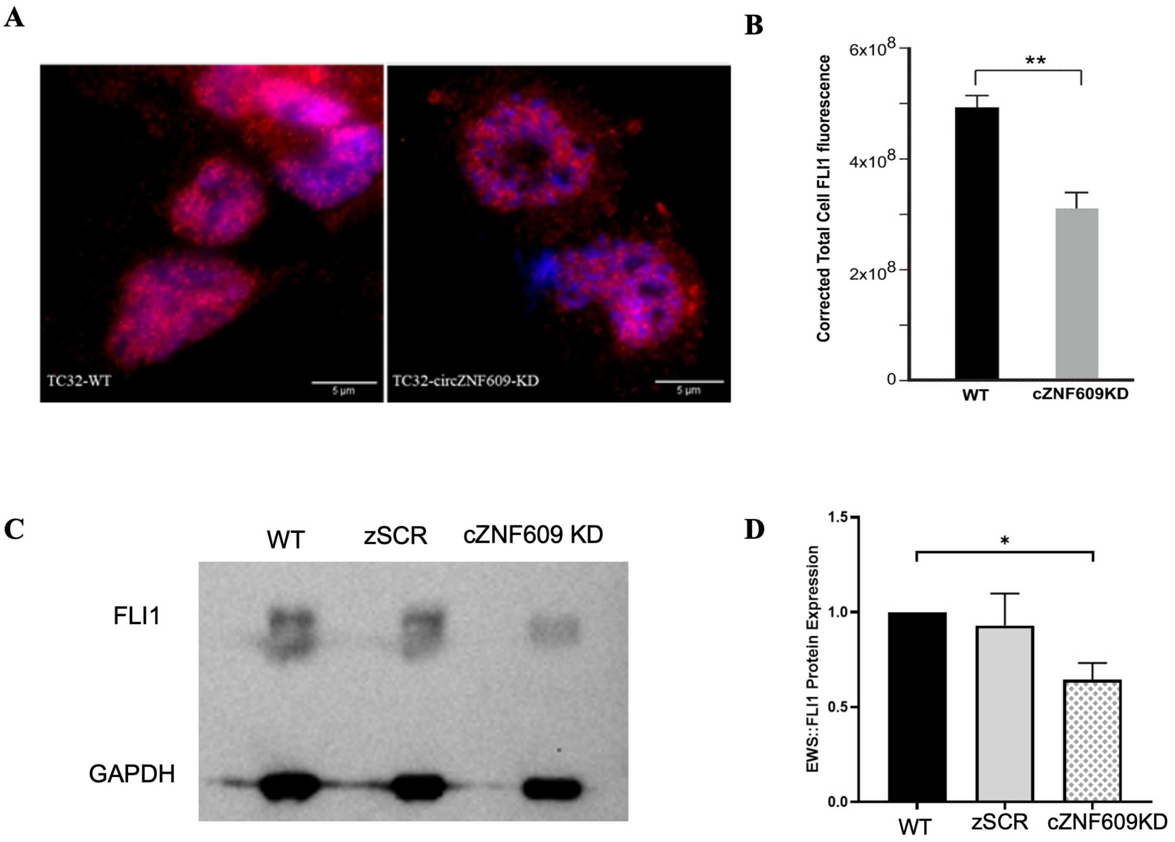

Figure S5: Downregulation of EWS::FLI1 protein levels in circZNF609 KD cells: A. Representative images of TC32 (WT and circZNF609-KD) cells after immunofluorescence staining. Raw merged z stacks of cells hybridized with FLI1 primary antibodies tagged with secondary AF647 antibodies (red) overlaid on DAPI (blue). The scale bar is 5 µm. **B.** Corrected total cell fluorescence (CTCF) of FLI1 protein (representing EWS::FLI1) in WT and circZNF609 KD in TC32 cells. The error bars indicate the 95% confidence interval for at least 70 cells. **C**. Western blot analysis for EWS::FLI1 protein expression across TC32 (WT, ZSCR, and circZNF609-KD) cells using anti-FLI1 antibody, GAPDH as loading control. **D.** Bar graph showing EWS::FLI1 protein expression levels from western blot across TC32 cells (WT, ZSCR, and circZNF609-KD). The error bars indicate the standard deviation between triplicates. * Indicates a significant difference with a p-value < 0.05, * *Indicates a significant difference with a p-value < 0.005.

Figure S6

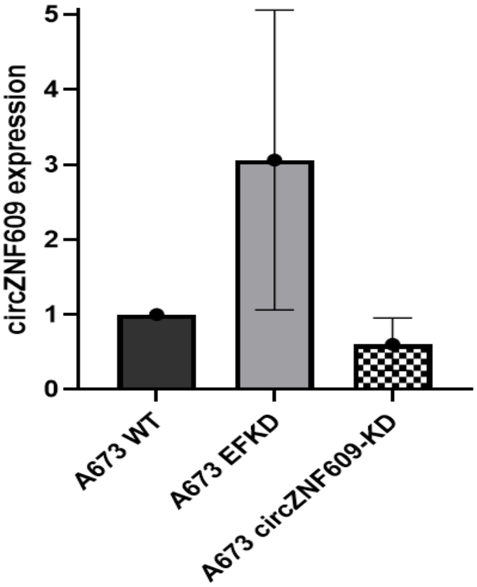

Figure S6: CircZNF609 associated peptide expression in EwS cells: qRT-PCR data showing circZNF609 levels in the polysome fraction of A673 WT, EF-KD and circZNF609-KD cells. The error bars indicate the standard deviation between triplicates.

Table S1: shRNA Sequences for circZNF60 Knockdown.

shRNA Target	Sequence
circZNF609	CCGGGTCTGAAAAGCAATGATGTTGCTCGAGCAACATCATTGCTTTTCAGAC TTTTTG

Table S2: Oligo Sequences for ZNF609 PC and PL CircFISH Probes Sets.

Probe Number	ZNF609 PL	ZNF609 PC
1	caggtgtattactgttggga	caatgatgagattccctact
2	ggtgctctgaagaattctgg	ttttccagatcggcgtccag
3	tgacatcctcagaagtgctg	tgacatttccagtttctgct
4	tattagctgaggaagggctg	gtatccccacctcctttgag
5	gaaaggggtttgtttttccg	tgtggccacagcattgggag
6	ctctgagctagaattcagct	caaacttgatgttgtctggt
7	tgagcccatggaattagtac	cttgaggacctggcactggg
8	tgtgcttgtacttcttgttg	gattttgatttgccttcctt
9	tggtggtacttaagtccatt	tttgccactcttactccttt
10	atcatctgtatgggcatgag	ctggagtgggtttgctagtg
11	atggagaataggttcctctc	tcacttggagtgaacaggga
12	ggctctacaagtcgaacttt	ctgcacctctttcttgctag
13	tttgcttgaaggagaagggc	cattggcaccatctcctgag
14	tttcttacagaggccttttg	ttgggagcaatagcagcaac
15	catcatcagagccatcattg	cttagccgccttctctgagc
16	ctgactttaggctcttggaa	ttttggaaccggctacactg
17	ggaaggtgtagatttgctgt	ttagatgagctgttctcctt
18	cagggttgactgtgaaaggt	cttcgcttctctccttcttg
19	ggactttcaagttcctttga	atccttttctgaacaagtcc
20	ctgaagattccctgaatggg	aagggaactggctggaggac
21	tctgatgagccatttaggag	tgcacttccatcatactgac
22	gtccgtgaaactgtagatct	tccacagctcctgtatccac
23	tagtgttttcaaggcggcta	ctcaatagctatactcccaa
24	tgttggttttgactgagctg	tagttcccaaaggattgagc
25	gagatgtcagagtatgcagg	cgacactcattctccccttc
26	tctttaaccaactgttcggc	agacttgactttctttagca
27	ggctgaggggaaaaaagagt	gtggacaacatcattgcttt
28	catgtacatattgggacgct	cacattggtcagtacatgcc
29	tactggttgtagtacaggga	tcagctatgttctcagacct
30	cagaaggtgggtgtggtaac	cccagctttcctattttcag
31	ttctgctgttcttcgtactg	aagattcaaggctcttcctt

32	gaattgacggcttttgcttc	tcttagagtcaacgtcccac
33	tgatgactgatttggctggg	gaggctccactgctcaagga
34	catcactcagcttcacttta	ggtttgcatccactcctttc
35	taccagagtattggatccat	ccactgtcgtatgtctcaac

Table S3: List of Primers used

Target Name	Forward Primer	Reverse Primer
GAPDH	TCGGAGTCAACGGATTTGGT	TTCCCGTTCTCAGCCTTGAC
ACTIN	TTGTTACAGGAAGTCCCTTGCC	ATGCTATCACCTCCCCTGTGTG
EWS::FLI1	GCAGCCTCCCACTAGTTACC	TCCCCTCCCAGGTGATACAG
CircZNF609	TGAGTGTCGCCTGCTAAAGA	CCCCCAGCTTTCCTATTTTC
Linear ZNF609	AATCTTGAGGTGGGACGTTG	TCCAGTTTCTGCTGGTCCTT
MiR-3667-3p	ACCTTCCTCTCCATGGGTCTTT	
MiR-1260a	ATCCCACCTCTGCCACCA	
MiR-3135b	GGCTGGAGCGAGTGCAGTGGTG	
MiR-3652	CGGCTGGAGGTGTGAGGA	
MiR-4755-5p	TTTCCCTTCAGAGCCTGGCTTT	
MiR-3145-3p	AGATATTTTGAGTGTTTGGAATTG	
MiR-145-5p	GTCCAGTTTTCCCAGGAATCCCT	
U6	Takara Bio Kit	Takara Bio Kit
3' MRQ Reverse	Takara Bio Kit	Takara Bio Kit

Table S4: List of antibodies used

Antibody Name	Catalog	Details
**Primary Antibodies**		
Mouse Anti-Human FLI1	BD Pharmingen, San Diego, CA, USA, 554266	Mouse monoclonal antibody raised against human FLI1, (Dilution: 1:700 in Blocking Buffer), Overnight incubation @ 4c.
GAPDH Loading Control Monoclonal Antibody (GA1R)	Invitrogen, Waltham, MA, USA, MA5-15738	Mouse monoclonal antibody raised against GAPDH (multiple species), (Dilution: 1:2000 in Blocking Buffer), Overnight incubation @ 4c.
Secondary Antibodies		
Rabbit anti-Mouse IgG (H+L) Secondary Antibody, HRP	Invitrogen, Waltham, MA, USA, 31450	Rabbit anti-mouse polyclonal secondary antibody, HRP conjugated, (Dilution: 1:20000 in Blocking Buffer), RT incubation for 1-2 hours.
